# Development, reach, acceptability and associated clinical changes of a group intervention to improve caregiver‐adolescent relationships in the context of adolescent depression

**DOI:** 10.1002/jcv2.12168

**Published:** 2023-05-23

**Authors:** Madison Aitken, Ameeta Sagar, Darren Courtney, Peter Szatmari

**Affiliations:** ^1^ Cundill Centre for Child and Youth Depression Centre for Addiction and Mental Health Toronto Ontario Canada; ^2^ Department of Psychiatry University of Toronto Toronto Ontario Canada; ^3^ Department of Psychiatry Hospital for Sick Children Toronto Ontario Canada

**Keywords:** adolescent, depression, intervention, parent‐child relationships, therapy

## Abstract

**Background:**

Adolescents with depression often experience relationship problems with their caregivers, which predict poorer treatment outcomes. Personalising interventions by targeting factors associated with poor treatment outcomes may enhance the effectiveness of interventions. We report the development and initial evaluation of an intervention designed to target caregiver‐adolescent relationship problems in the context of adolescent depression.

**Methods:**

Following a literature search to identify established caregiver interventions, we developed a new group intervention for caregivers through an iterative process including six rounds of the group with *n* = 53 caregivers of adolescents age 13–18 in the context of an integrated care pathway for adolescent depression. Caregivers rated their family functioning at the beginning and end of the program and provided anonymous satisfaction ratings. Enrolment and attendance data were examined. Youth with lived experience of depression and their caregivers provided input that was incorporated in the final version of the intervention.

**Results:**

The final intervention consists of 8 weekly, 1.5 h group sessions, delivered face‐to‐face, addressing: psychoeducation, the cognitive‐behavioural model and caregiving, positive caregiving, listening and validation, expressing emotions effectively, and problem solving. Reach (56%), attendance (*M* = 63%, *SD* = 31%), and satisfaction (*M* = 92%; *SD* = 7%) supported the feasibility of the program. Caregivers reported significant improvements in family functioning, *t*(21) = 2.68, *p* = .014, *d*_*z*_ = 0.56 [95% CI 0.11–1.0].

**Discussion:**

A group intervention is acceptable to caregivers of adolescents with depression and may be associated with improved family functioning. Further research is needed, including a randomised controlled trial to test effects of the intervention on various dimensions of the caregiver‐youth relationship and on youth depression outcomes.

## Graphical abstract

Caregiver‐adolescent relationship problems are associated with poorer response to treatment for adolescent depression. We developed a caregiver group intervention targeting caregiver‐adolescent relationship problems through a multi‐step process incorporating theory and input from youth with lived experience of depression and their caregivers. The program was acceptable to caregivers based on reach, satisfaction, and attendance, and caregivers reported significant improvements in family functioning.

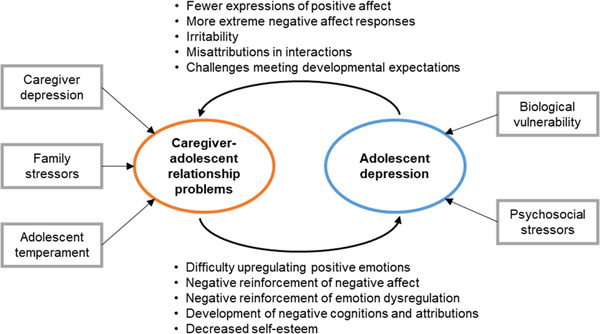

**Key points**

- Caregiver‐adolescent relationship problems are associated with poorer response to treatment for adolescent depression.
- We developed a caregiver group intervention targeting caregiver‐adolescent relationship problems through a multi‐step process incorporating theory and input from youth with lived experience of depression and their caregivers.
- A group intervention targeting caregiver‐adolescent relationship problems was acceptable to caregivers based on reach, satisfaction, and attendance, and caregivers reported significant improvements in family functioning.
- Further research is needed to test effects on caregiver‐adolescent relationship problems in a randomised controlled trial.

## INTRODUCTION

One in 10 adolescents experience a major depressive episode, increasing their risk of school dropout, academic, family, and social impairment and carrying a 16 times greater likelihood of suicidal ideation compared to their peers (Avenevoli et al., 2015; Fletcher, 2010; Glied & Pine, 2002). Evidence‐based treatments for adolescent depression (medication and psychosocial) have only small‐to‐moderate effects (Weisz et al., 2006) and many adolescents do not respond sufficiently (Davies et al., 2020) or experience depression recurrence following treatment (Curry et al., 2011). There is increasing recognition that a personalised approach targeting risk and perpetuating factors in the adolescent's environment, such as the caregiver‐adolescent relationship, is needed to improve depression outcomes (Ng & Weisz, 2016; Weisz et al., 2018).

Although conflict with parents was previously viewed as part of typical adolescent development, high levels of conflict are not normative and are often present alongside adolescent mental health problems (Steinberg, 2001). Relationship problems between caregivers^1^ and adolescents are especially common when adolescents experience depression (Alaie et al., 2020; Schwartz et al., 2017). Secondary analyses of clinical trials suggest that caregiver‐adolescent relationship problems may be a significant factor that limits adolescent response to evidence‐based treatments for depression (Birmaher et al., 2000; Brent et al., 2009; Gunlicks‐Stoessel et al., 2010; Wilkinson et al., 2011). A recent review of clinical trials for adolescent depression found that of 53 predictor and moderator variables tested, parent‐child conflict was among the variables most consistently associated with less favourable outcomes (Courtney, Watson, et al., 2022).

The association between caregiver‐adolescent relationship problems and adolescent depression is multifactorial and subject to bidirectional influences (Hale et al., 2016; Miklowitz, 2004; Rengasamy et al., 2013). Changes in mood and behaviour associated with a depressive episode in the adolescent (e.g., irritability, poor school attendance) may elicit frustrated and/or overprotective reactions from caregivers, which may contribute to worsening caregiver‐adolescent relationships (Coyne et al., 1991; Miklowitz, 2004; Steinberg, 2001). Caregivers' behaviour (e.g., invalidation, lack of acceptance) may also affect adolescents' risk for depression by creating cycles of increased youth negative affect (Miklowitz, 2004; Shenk & Fruzzetti, 2011) and contributing to the development of low self‐esteem and cognitive biases (Garber & Flynn, 2001). Interventions that reduce negative caregiver‐adolescent interactions and increase adaptive communication may interrupt reciprocal associations between adolescent depression and caregivers' reactions, potentially leading to more positive interactions that could support recovery from depression and reduce depression recurrence. However, no existing interventions have demonstrated efficacy for reducing the caregiver‐adolescent relationship problems commonly present in adolescents with depression (National Institute for Health and Clinical Excellence [NICE], 2019).

### Current approaches to improving caregiver‐adolescent relationships

We reviewed studies included in previous systematic reviews and meta‐analyses (David‐Ferdon & Kaslow, 2008; Kaslow & Thompson, 1998; Weersing et al., 2017) to determine how caregivers have been involved in interventions for adolescent depression and to identify existing interventions for caregivers of adolescents with depression and conducted further searches of studies published after 2014 (see supporting information for search strategy).

Our search identified one caregiver intervention (Coping with Depression‐Adolescent [CWD‐A]; CWD‐A Parent program), which showed limited effects on adolescent depression and was not designed to address high levels of caregiver‐adolescent conflict; instead, the program included an equal emphasis on basic communication strategies and on teaching caregivers about the cognitive behavioural therapy strategies adolescents were learning (Clarke et al., 1999; Lewinsohn et al., 1990).

Other caregiver‐adolescent relationship interventions have limitations that may make them unsuitable in the context of adolescent depression. For example, family therapy (i.e., therapy in which parents and available siblings participate along with the adolescent, and which focuses on changing interaction patterns and relationships between all family members; Liddle, 2002; Miklowitz & Chung, 2016) is less acceptable to adolescents than other depression treatments, potentially because of adolescents' desire for autonomy from parents (Caporino & Karver, 2012). Family therapy is also resource‐intensive (Goorden et al., 2016), and is less effective than other treatments for adolescent depression (Brent et al., 1997), with effects on adolescent depression and self‐harm comparable to usual care (Cottrell et al., 2018; Diamond et al., 2019). Other approaches (Fosco et al., 2016) are primarily preventive and may not be sufficient for clinic‐referred adolescents. Finally, problem‐solving interventions have been effective in families of adolescents with disruptive behaviour disorders (Barkley et al., 2001), but their effectiveness has not been tested in the context of adolescent depression.

Previous trials have shown that group interventions for caregivers and family members of adults with depression or schizophrenia, adolescents with substance use disorders, or children with low self‐confidence, are associated with improved communication with their child or family member and have effects comparable to family therapy (Friedman, 1989; Graybill, 1986; Katsuki et al., 2011; Leff et al., 1990; Shimazu et al., 2011). Moreover, decades of research in the field of disruptive behaviour has shown that caregiver‐focused interventions are effective in changing caregivers' behaviour (Dekkers et al., 2022; Forgatch & Patterson, 2010) and that increasing adaptive caregiver behaviour initiates positive cascade effects that predict favourable youth mental health outcomes (Patterson et al., 2010). Therefore, a group intervention focused on improving caregiver‐adolescent relationships may allow clinicians to personalise care by addressing the relationship problems present for many adolescents with depression and their caregivers.

### Present project

The purpose of this project was to develop a caregiver intervention that may improve caregiver‐adolescent relationships and support adolescent recovery from depression. This intervention was developed to address the following: (1) lack of evidence‐based approaches to involving caregivers when adolescents are in treatment for depression (National Institute for Health and Clinical Excellence [NICE], 2019); (2) the need to better incorporate caregivers to improve depression treatment outcomes in adolescents (James Lind Alliance, 2018); and (3) the need to improve caregivers' ability to support youth with mental health problems, including responding in ways that do not increase youth distress (James Lind Alliance, 2018; Midgley et al., 2015; Stapley et al., 2016).

We report our intervention development process following the Guidance for the Reporting of Intervention Development (GUIDED; see Table S2) checklist (Duncan et al., 2020). Our report includes evidence gathered through the intervention development process from caregiver participants in pre‐pilot tests of the program, including reach of the program, session attendance and drop‐out, satisfaction ratings, and changes in self‐reported family functioning. We describe the resulting intervention following the Template for Intervention Description and Replication (TIDieR; see Table S3; Hoffmann et al., 2014).

## MATERIALS AND METHODS

### Context and target population

The intervention was developed in an outpatient child and youth treatment program in a publicly funded hospital in a large, diverse Canadian city as part of an integrated care pathway for adolescent depression based on established guidelines for the treatment of youth depression (Courtney et al., 2019; NICE, 2019). All adolescents had been referred for outpatient mental health services and had been diagnosed with major depressive disorder. In addition, adolescents in the care pathway for depression had high rates of comorbid generalised anxiety disorder (80%) and anti‐depressant medication use (63%; Courtney, Cheung, et al., 2022).

The integrated care pathway included initial caregiver and adolescent orientation and psychoeducation sessions, measurement‐based care, and optional adolescent psychotherapy (group cognitive behavioural therapy, or other psychotherapy) and medication (Courtney et al., 2019). The present intervention is intended for adult caregivers of an adolescent age 13–18 with prominent depression symptoms that have been identified as a treatment target by the adolescent and their clinician, when problems in the caregiver‐adolescent relationship are identified by either adolescent or caregiver.

### Intervention development approach and guiding principles

Intervention development followed the steps recommended for complex interventions by the Medical Research Council (Craig et al., 2008), including: (1) identifying existing evidence; (2) identifying and developing theory; and (3) modelling process and outcomes. The process was guided by two key principles: (1) the benefits of delivering caregiver interventions in a group format, including being resource‐efficient and offering caregiver‐to‐caregiver peer support (Kaminski & Claussen, 2017); and (2) the need for the intervention to be acceptable to caregivers, adolescents, and clinicians.

### Use of components from existing interventions

The intervention includes a small number of components drawn from two existing interventions (see Table 2): (1) CWD‐A program Parent Group (Lewinsohn et al., 1991); and (2) Care for Adolescents who Receive Information ‘Bout OUtcomes (CARIBOU) Group Cognitive Behavioural Therapy program (Courtney et al., 2019).

### Use of theory to inform intervention development

**FIGURE 1:**
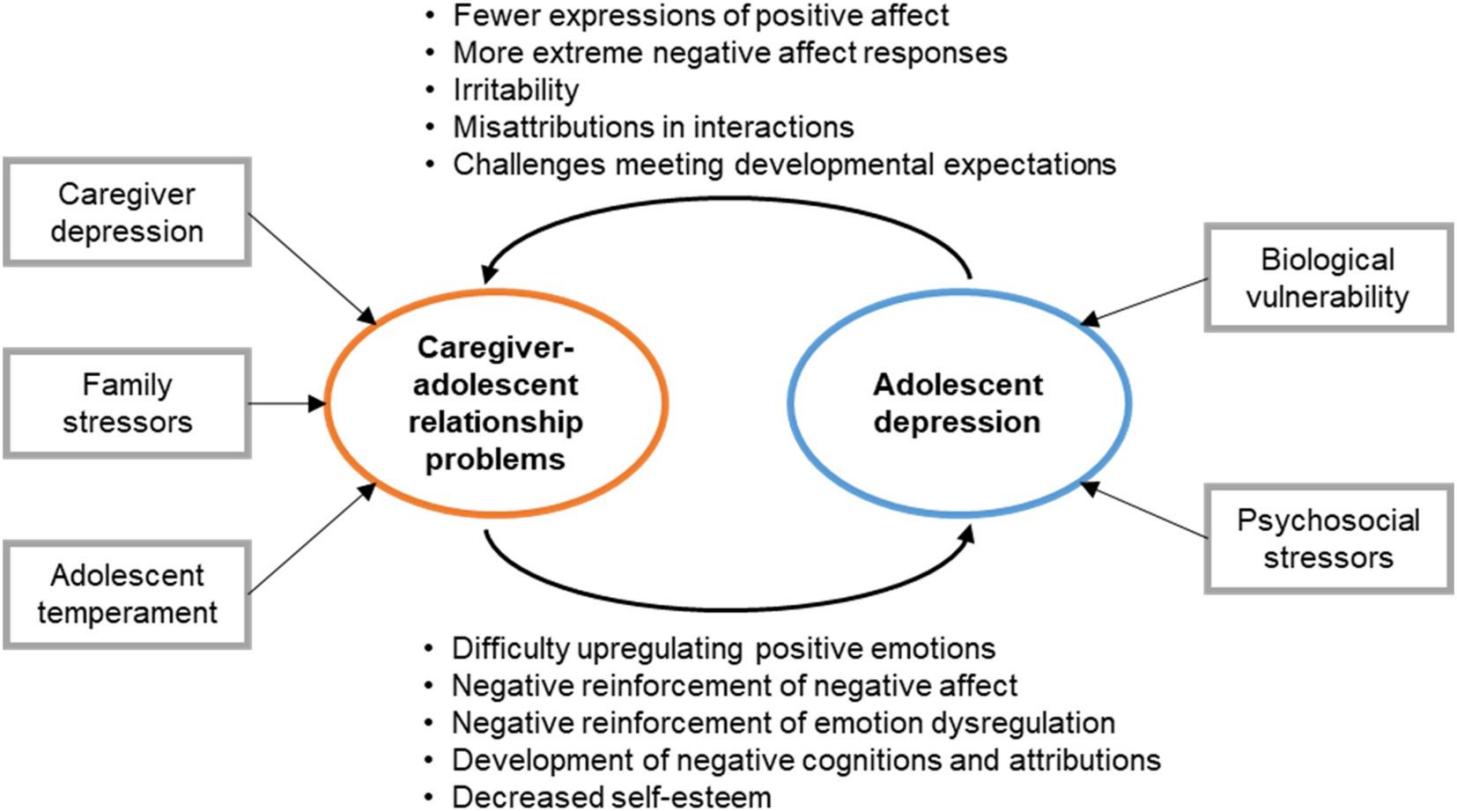
Theoretical model of bidirectional associations between caregiver‐adolescent relationship problems and adolescent depression.

We developed a theoretical framework (Figure 1) integrating biopsychosocial perspectives on caregiver‐youth relationships and mental health (Beauchaine, 2015; Miklowitz, 2004) and evidence of bidirectional associations between caregiver‐youth relationships and mental health (Coyne et al., 1991; Garber & Flynn, 2001; Katz & Hunter, 2007; Rengasamy et al., 2013; Schwartz et al., 2012; Sheeber et al., 2012), which informed the intervention content. In brief, caregiver‐youth relationship problems and youth depression are seen as contributing to one another (Hale et al., 2016), in addition to each having their own risk factors. Additional risk factors for adolescent depression include biological vulnerabilities and psychosocial stressors (Abela & Hankin, 2008). Additional risk factors for caregiver‐adolescent relationship problems include caregiver depression (Frye & Garber, 2005), family stressors (Schleider & Weisz, 2017), and adolescent temperament (Lougheed, 2020). Caregiver‐adolescent relationship problems may increase risk for adolescent depression through multiple pathways, including an increased likelihood for adolescents to have negative cognitions about themselves, decreased self‐esteem, and negative reinforcement of dysregulated negative affect (Beauchaine, 2015; Garber & Flynn, 2001). On the other hand, adolescent depression may contribute to worsening caregiver‐adolescent relationships through increased adolescent irritability (Alaie et al., 2020), increases in negative attributions for caregivers' behaviour (Rote et al., 2021), and an increase in points of conflict when adolescents are not meeting developmental expectations such as attending school (Nelemans et al., 2014).

Our review of the literature identified three indicators of caregiver‐adolescent relationship problems consistently associated with adolescent depression onset, severity, and treatment response. First, greater caregiver *expressed emotion* (Hooley & Parker, 2006) is associated with more youth depression symptoms cross‐sectionally (McCleary & Sanford, 2002), increases in depression symptoms over time (Frye & Garber, 2005), and poorer youth depression treatment outcomes (Asarnow et al., 1993). Second, maladaptive *affective responding* (Katz & Hunter, 2007) is associated with higher levels of adolescent depression (Katz et al., 2014; Shortt et al., 2016; Yap et al., 2008) and predicts the development of depression in prospective longitudinal samples (Nelis et al., 2019; Schwartz et al., 2014). Third, *caregiver‐adolescent conflict* is associated with more adolescent depression symptoms cross‐sectionally and over time (Sheeber et al., 1997, 2007; Weymouth et al., 2016) and predicts poorer response to evidence‐based treatment for adolescent depression (Courtney, Watson, et al., 2022). Bidirectional associations between each of these three relationship indicators and adolescent depression symptoms have been identified (Coyne et al., 1991; Hale et al., 2016; Nelis et al., 2019). Intervention content was developed to ensure these three key aspects of relationship problems were addressed.

### Use of evidence to inform intervention development

**FIGURE 2:**
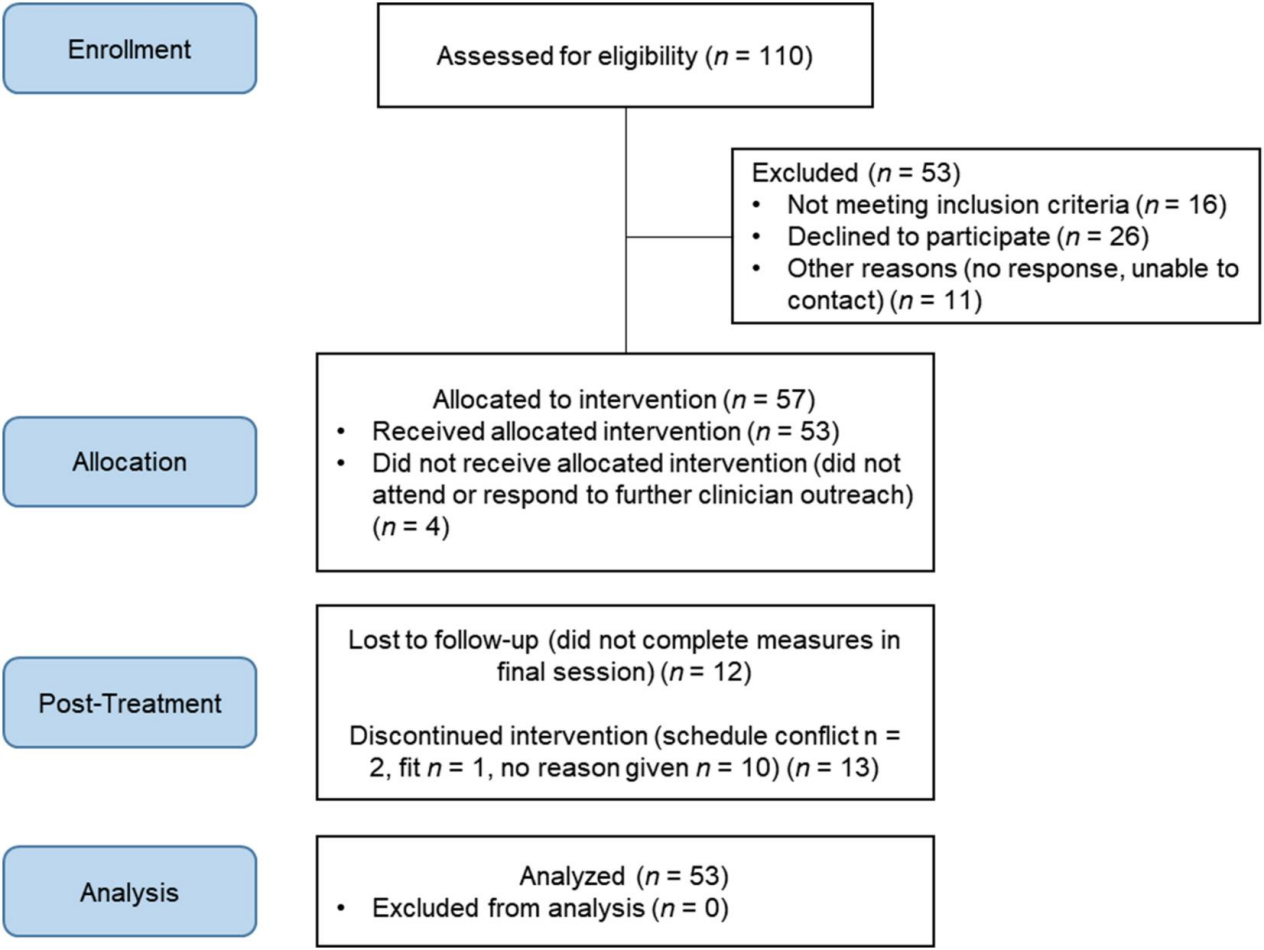
CONSORT diagram showing flow of caregiver participants through recruitment and follow‐up.

We carried out six rounds of the group (*N* = 53 caregivers of 41 adolescents; see Figure 2 for CONSORT diagram) in a single‐arm, non‐masked feasibility trial, during which we measured: (1) reach (number of caregivers who participated, and reasons for declining to participate); (2) drop out (≥3 missed sessions) and attendance; (3) caregiver satisfaction(see details below); and (4) caregiver ratings on the McMaster Family Assessment Device (MFAD) General Functioning scale (Epstein et al., 1983), consisting of the mean of 12 items, each rated on a 4‐point scale, completed at the beginning and end of the group program (used as a proxy for caregiver‐adolescent relationship problems).

Caregiver satisfaction was measured through anonymous ratings completed in the final group session on a locally‐developed scale containing items about the group content, facilitation, and program quality (e.g., *Overall, how satisfied are you with the group?*), each rated on a 4‐point scale. This survey was collected as part of routine clinical practice and therefore the items varied slightly over time (see supporting information for details and for the questionnaire version used in three of the six rounds). We created a satisfaction percentage score by summing the item ratings and dividing by the maximum possible score for that questionnaire version.

Descriptive statistics were examined for reach (percent of caregivers contacted who agreed to participate and reasons for not participating; Glasgow et al., 2019), drop out, attendance, and satisfaction. Change in family functioning was examined using a paired samples *t*‐test in caregivers with data available at both time points (as we did not have sufficient additional information available to impute missing data). Effect size (Cohen's *d*_z_ with Hedge's correction for small sample size) for change in family functioning was calculated based on the standardised mean difference without controlling for the correlation between measures (Lakens, 2013).

### Stakeholder contributions

#### Caregivers

Group facilitators solicited feedback from all caregivers who participated in the program. Facilitators conveyed in the initial session that the group was in development and that caregivers' feedback would be used to improve future iterations of the group. Caregivers provided written feedback in the final group session through open‐ended questions on the anonymous satisfaction questionnaire (e.g., *Please share your suggestions for improving our parent/caregiver group*; see Supporting Information S1). Caregivers also provided feedback about their experience to facilitators orally throughout the group and in the final session. Caregiver feedback was reviewed by group facilitators and incorporated into subsequent sessions and iterations of the group.

Once a stable version of the intervention was developed, two caregivers who had previously participated in the group were recruited as paid advisors. These caregivers reviewed the session outlines and handouts and provided written and oral feedback on ways to improve the content and delivery of the group.

#### Youth

Adolescents with lived experience of depression (2) were recruited as paid advisors along with an experienced Youth Engagement Facilitator, who also provided input on the intervention content. Youth advisors reviewed session outlines and handouts and provided feedback in writing and through a series of collaborative meetings with the first author. See supporting information (Table S1) for a summary of changes made based on caregiver and adolescent feedback.

A list of feedback from caregiver and youth stakeholders and resulting changes to the program content is included in the Supporting Information S1.

## RESULTS

### Reach

A total of 53 caregivers (aged 27–62) of 41 adolescents (aged 14–18) participated in the group during the intervention development process, representing a 56% reach (*N* eligible = 94).

Reasons eligible caregivers (*n* = 41) did not participate included schedule conflict (44%, *n* = 18), no response or unable to contact caregiver (27%, *n* = 11), distance to travel was too great (10%, *n* = 4), not interested (7%, *n* = 3), no reason given (2%, *n* = 1), and agreed to participate but did not follow through (10%, *n* = 4). Sample descriptive statistics are presented in Table 1.

**TABLE 1:** Sample descriptive statistics.

Variable	% or *M* (*SD*)
Caregiver age	50.2 (6.8)
Caregiver type	
Father	28
Mother	70
Adult sibling	2
Youth age	16.0 (1.1)
Youth gender	
Boy	40
Girl	58
Nonbinary	2
Family functioning^a^	2.20 (0.46)

^a^ Baseline score; Scores ≥2.0 are in the clinical range.

### Acceptability and changes in family functioning

Caregivers who participated attended an average of 65% of sessions (*SD* = 29.1%), mode = 7 sessions, *M*(*SD*) = 5.3 (2.4). In terms of dropout, 75% of caregivers who began the intervention completed it, whereas 25% were considered to have dropped out.

**FIGURE 3:**
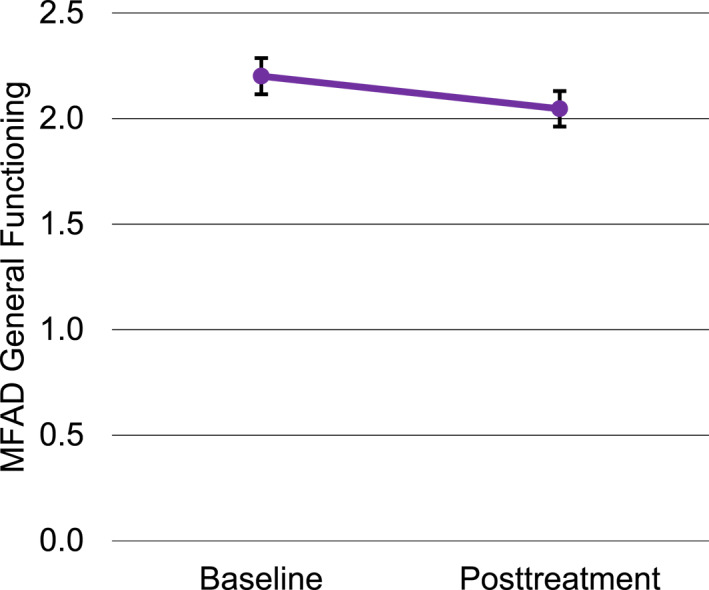
Change in caregiver‐rated family functioning from baseline to posttreatment (error bars represent standard error).

There were 34 caregivers with satisfaction ratings from the final session of the group. Caregiver satisfaction ranged from 75% to 100% (*M* = 92%; *SD* = 6.8%). There were 29 caregivers who completed ratings of family functioning in the first group session (baseline) and 30 who completed ratings in the final session. Missing data on the family functioning scale was primarily due to caregivers being absent from the first or last session, during which the ratings were completed. Figure 3 presents mean family functioning scores at baseline and post‐treatment for all participants with available data. At baseline, ratings of family functioning were in the clinical range for 66% of families based on established cut‐offs for the MFAD General Functioning Scale (Miller et al., 1985). Among those with ratings at both time points (*n* = 22), family functioning problems decreased significantly from baseline (*M* = 2.16, *SD* = 0.48) to the final session (*M* = 2.05, *SD* = 0.47, *t*(21) = 2.67, *p* = .014, *d*_*z*_ = 0.56 [95% CI 0.110–0.997]).

### Description of the final intervention program

The final intervention is a group program for caregivers that targets the communication challenges commonly present when adolescents experience depression. It consists of eight face‐to‐face group sessions, each 1.5 h long. Each session includes a brief review of the previous session, review of home practice, introduction and discussion of a new topic, in‐session practice, and assignment of home practice. Each session has an accompanying handout summarising key points with space to track home practice. The intervention is designed to be delivered by two facilitators with training and experience in cognitive behavioural therapy and in providing mental health care to adolescents and caregivers. Table 2 shows the content covered in each session. Dissemination plans for the manual are currently being developed.

**TABLE 2:** Content of the caregiver intervention by session.

Session	Topic	Content
1	Introduction and psychoeducation about depression	Orientation and icebreakers Depression in young people^b^ Cognitive‐behavioural triangle Downward and upward mood spirals^a^ Setting goals
2	Cognitive‐behavioural model & caregiving	Cognitive‐behavioural model in relation to caregiving Accommodating and authoritative caregiving
3	Positive caregiving strategies	Supporting upward mood spirals Positive caregiving strategies Natural and logical consequences
4	Communication part 1—Receiving skills	How depression affects communication Communication as a skill^a^ Active listening^a^ and validation
5	Communication part 2—Sending skills	Rules for effective self‐disclosure^a^ Sharing pleasant feelings^a^ Sharing difficult feelings^a^
6	Problem solving—Part 1	Introducing problem solving steps^b^ Step 1: Defining the problem^b^
7	Problem solving—Part 2	Additional problem solving steps^b^ Problem solving practice
8	Review and maintenance	Review of program content Discussion of other issues and challenges Planning for future challenges

^a^ Indicates content adapted from the Coping with Depression‐Adolescent (CWD‐A) Parent Program (Lewinsohn et al., 1991).

^b^ Indicates content adapted from the Care for Adolescents who Receive Information ‘Bout OUtcomes (CARIBOU) program (Courtney et al., 2019).

## DISCUSSION

Treatment of adolescent depression remains a clinical challenge, and many adolescents do not benefit sufficiently from evidence‐based interventions, or experience recurrence of depression following treatment (Weisz et al., 2006). We have developed an intervention designed to target caregiver‐adolescent relationship problems, which appear to be a major factor that limits treatment benefit (Courtney, Watson, et al., 2022). The intervention was developed through an iterative process, consisting of literature searches, theory development, content development by expert clinicians, piloting and gathering caregiver feedback, revising, and obtaining input from youth with lived experience of depression and their caregivers. As a result, we believe this program may be useful in improving caregiver‐adolescent relationships in the context of depression. Our preliminary data suggest that the intervention is acceptable to caregivers, having good reach (based on the proportion of caregivers who agreed to participate, and the similarity of reasons caregivers were unable to join to those cited by other caregivers in our clinical program) and high satisfaction ratings, and that participation may be associated with reductions in family conflict (used as a proxy for caregiver‐youth relationship problems).

### Potential changes needed for tailoring

It is important that caregivers feel that the content of the group reflects their experience. In delivering the caregiver group intervention in other contexts, facilitators may wish to make adjustments to meet the needs of the caregivers in the group. Tailoring that may increase caregiver engagement includes the use of updated or adapted examples; inclusion of a youth or caregiver co‐facilitator with lived experience; and use of open dialogue and questions to understand the fit of the content with the experiences of caregivers from diverse cultural backgrounds.

### Remaining uncertainties and future directions

The caregiver group intervention was designed to be delivered as part of a care pathway for adolescent depression (Courtney et al., 2019) in a hospital outpatient mental health program and has not been tested outside this context. Since developing the intervention, we have run two rounds of the group via secure videoconference. Previous studies have demonstrated the acceptability of internet‐delivered group parent interventions (Douma et al., 2020; Kuhlthau et al., 2020) and their potential to reduce common barriers to participation, such as geographic location, transportation, and childcare (Tully et al., 2017). Indeed, over half of the eligible caregivers who did not participate in our in‐person groups cited schedule constraints or travel distance. Formal evaluation of the internet‐delivered version is needed to measure caregiver satisfaction and attendance in this modified format. Finally, clinician fidelity was not assessed because the intervention was in development, and future research is needed to measure facilitator adherence to the content.

We found a small but significant improvement in family functioning over the course of the 8‐week program. Factors that may have contributed to the effects being small include measuring change in overall family functioning (whereas the intervention targeted only caregiver‐adolescent communication), the short time frame in which change was measured, clinical levels of parent‐adolescent relationship problems not being required for inclusion, and the fact that the intervention was still in development. Further research is needed to determine the effects of the caregiver intervention on a comprehensive set of indicators of caregiver‐adolescent relationship quality important for adolescent depression (expressed emotion, affective responding, and conflict) and on adolescent depression treatment outcomes. Lastly, given the high rates of depression among caregivers of adolescents with depression (Weissman, 2016), it will be important to test caregiver depression as a moderator or mediator of intervention effects.

### Limitations

Our results should be considered in the context of several limitations. First, caregivers only participated in the intervention if they were involved in their adolescent's care in some way. It is possible that caregivers having highly problematic relationships with their adolescent were not involved in their adolescent's mental health care (as permitted under our provincial health care consent regulations); therefore, some caregivers with highly problematic relationships with their adolescent may not have been informed about the study. Second, our analysis of intervention effects on family functioning included only a relatively small number of caregivers with complete data and did not use a comparison condition; therefore, these results should be interpreted cautiously given the potential for bias, including placebo effects and regression to the mean. Third, we used a measure of overall family functioning as a proxy for caregiver‐adolescent relationship problems, and further research measuring change in the caregiver‐adolescent dyadic relationship are needed to draw firmer conclusions. Finally, program reach may differ if delivered in other contexts, including when the program is presented as a consolidated program, versus the present initiative, which focused on developing the intervention.

## CONCLUSION

We have developed a group intervention for caregivers designed to address caregiver‐adolescent relationship challenges often present when adolescents experience depression. Our results suggest that caregivers find the intervention acceptable, that they perceived improvements in their family functioning by the end of the group, and that the program has the potential to reach the majority of eligible caregivers, most of whom complete the program and attend the majority of sessions. The present intervention warrants further testing in a randomised controlled trial to determine its effects on caregiver‐adolescent relationship problems in the context of adolescent depression.

## AUTHOR CONTRIBUTIONS

**Madison Aitken**: Conceptualization; Data curation; Formal analysis; Funding acquisition; Investigation; Methodology; Project administration; Writing – original draft; Writing – review & editing. **Ameeta Sagar**: Conceptualization; Methodology; Project administration; Resources; Writing – review & editing. **Darren Courtney**: Conceptualization; Methodology; Writing – review & editing. **Peter Szatmari**: Conceptualization; Funding acquisition; Resources; Writing – review & editing.

## CONFLICT OF INTEREST STATEMENT

The authors have declared they have no competing or potential conflicts of interest.

## ETHICAL CONSIDERATIONS

This project used de‐identified clinical data without written consent and was approved by our hospital Research Ethics Board.

## Supporting information

Supporting Information S1

## Data Availability

The data are not available due to privacy or ethical restrictions.

## ENDNOTE

^1^
We use the term ‘caregiver’ throughout, which includes parents and other adults who may be responsible for adolescents' wellbeing.
