# Supplementary material for: Development, reach, acceptability and associated clinical changes of a group intervention to improve caregiver‐adolescent relationships in the context of adolescent depression
Source: JCPP Adv. 2023 May 23;3(4):e12168. doi: 10.1002/jcv2.12168 (PMC10694543; doi:10.1002/jcv2.12168)
Supplement: Supplementary file 1 — Supporting Information S1 [file JCV2-3-e12168-s001.docx]

**Supporting Information**

*Development, Reach, Acceptability and Associated Clinical Changes of a Group Intervention to Improve Caregiver-Adolescent Relationships in the Context of Adolescent Depression*

Madison Aitken, Ameeta Sagar, Darren Courtney, & Peter Szatmari

**Search Strategy for Identifying Current Approaches to Improving Caregiver-Adolescent Relationships**

Previous systematic reviews and meta-analyses on interventions for adolescent depression included studies published in 2014 or earlier. We therefore conducted an updated search in PsycINFO, applying the following inclusion criteria: 1) publication date after 2014; 2) peer-reviewed; 3) English language; 4) age group = adolescence; 5) methodology = clinical trial, treatment outcome, or empirical study. We also reviewed studies included in two related systematic reviews, one on cognitive-behavioural therapy for adolescent internalizing disorders (Sun et al., 2018) and one on parental involvement in adolescent depression interventions (Dardas et al., 2018).

**Changes to the Intervention over the Course of Development**

Feedback from caregiver group participants (through anonymous satisfaction surveys), as well as from paid caregiver and youth advisors, indicating suggestions for improving the program, is summarized in Table S1. Table S1 also contains information on the changes made to the program over the course of development in response to this feedback (Section A), and an explanation where changes were not made (Section B). Some of the changes made to the intervention over the course of the project based on feedback from youth and caregiver stakeholders: 1) increased relevance of examples used, to focus on areas caregivers and youth identified as priorities (e.g., an example about an adolescent not attending a mental health appointment was perceived as more relevant to youth and caregiver advisors than not helping with household chores); 2) enhanced psychoeducation about depression to match content delivered to adolescents in the depression care pathway about the core symptoms of depression, symptoms commonly associated with depression, and causes of depression (Courtney et al., 2021); 3) the addition of information for caregivers on how to respond to thoughts of suicide; 4) supplementing existing content on active listening with teaching and practicing validation; and 5) enhanced suggestions for caregivers regarding communication strategies that youth with depression may perceive as supportive. In addition, based on our ongoing review of literature identifying the dampening of positive affect as an important hallmark of caregiver-adolescent communication when adolescents experience depression, we added content on how to enhance adolescents’ positive affect and avoid dampening positive affect.

**Caregiver Satisfaction Ratings**

A sample caregiver satisfaction questionnaire is provided in Appendix A. The format of the satisfaction questionnaire varied across rounds of the group. In Round 1, 6 items were rated on a scale from *Not Helpful* to *Very Helpful*, and a single open-ended prompt was used: “*Comments/feedback.*” In Round 2, the 5 Likert-rated items shown in Appendix A were used, along with the 3 open-ended questions shown in Appendix A (but we did not include ratings of specific sessions/topics). In Rounds 3-6, caregivers also rated individual sessions/topics, as shown in Appendix A, though the specific wording varied as the content and organization of the program evolved across the intervention development process.

References

Courtney, D. B., Relihan, J., Darnay, K., & Ameis, S. (2021). *CARIBOU Mood Foundations facilitator guide: An educational intervention for adolescents with depression and their caregivers*. Centre for Addiction and Mental Health.

Dardas, L. A., van de Water, B., & Simmons, L. A. (2018). Parental involvement in adolescent depression interventions: A systematic review of randomized clinical trials. *International Journal of Mental Health Nursing*, *27*(2), 555–570. https://doi.org/10.1111/inm.12429

Duncan, E., O’Cathain, A., Rousseau, N., Croot, L., Sworn, K., Turner, K. M., Yardley, L., & Hoddinott, P. (2020). Guidance for reporting intervention development studies in health research (GUIDED): An evidence-based consensus study. *BMJ Open*, *10*(4), 1–12. https://doi.org/10.1136/bmjopen-2019-033516

Hale, W. W., Crocetti, E., Nelemans, S. A., Branje, S. J. T., van Lier, P. A. C., Koot, H. M., & Meeus, W. H. J. (2016). Mother and adolescent expressed emotion and adolescent internalizing and externalizing symptom development: A six-year longitudinal study. *European Child and Adolescent Psychiatry*, *25*(6), 615–624. https://doi.org/10.1007/s00787-015-0772-7

Hoffmann, T. C., Glasziou, P. P., Boutron, I., Milne, R., Perera, R., Moher, D., Altman, D. G., Barbour, V., Macdonald, H., Johnston, M., Kadoorie, S. E. L., Dixon-Woods, M., McCulloch, P., Wyatt, J. C., Phelan, A. W. C., & Michie, S. (2014). Better reporting of interventions: Template for intervention description and replication (TIDieR) checklist and guide. *BMJ (Online)*, *348*(March), 1–12. https://doi.org/10.1136/bmj.g1687

Stapley, E., Midgley, N., & Target, M. (2016). The experience of being the parent of an adolescent with a diagnosis of depression. *Journal of Child and Family Studies*, *25*(2), 618–630. https://doi.org/10.1007/s10826-015-0237-0

Sun, M., Rith-Najarian, L. R., Williamson, T. J., & Chorpita, B. F. (2018). Treatment features associated with youth cognitive behavioral therapy follow-up effects for internalizing disorders: A meta-analysis. *Journal of Clinical Child and Adolescent Psychology*, 1–15. https://doi.org/10.1080/15374416.2018.1443459

Table S1

*Summary of Youth and Caregiver Feedback, Changes Made, or Rationale where Changes Not Made*

1. **Feedback and Resulting Changes**

| Source | Feedback Theme and Description | Changes Made |
| --- | --- | --- |
| Caregivers | More time to connect with other caregivers: Caregivers valued opportunities to receive support from, and hear about experiences of, other caregivers, and wanted more time for this in group | - Increased the amount of opportunities for caregivers to engage with and support one another through group discussion |
| Caregivers | Managing time and discussion: Need to ensure that all parents have opportunities to participate, that no group participants monopolize the discussion, and sessions end on time | - Revised group rules to include facilitators moving discussion along and ensuring everyone has opportunities to participate - Facilitators ensured throughout that this and other group rules were being followed |
| Caregivers | Handouts useful but could be improved: Caregivers wanted to receive handouts before the session (e.g., by email), and as a package at the end of the program, and for handouts to be updated | - Handouts updated to reflect revised program content and be more visually appealing - Handouts sent to caregivers by email before each session and provided as a package at the end of the program |
| Caregivers | Additional topics or content: Caregivers wanted suggested materials for further reading following group, more role plays, and more strategies | - Provided a list of resources for additional reading - Added validation, in addition to existing active listening content Added more role plays, especially in validation and problem solving sections |
| Caregivers | Caregiver stress management: Incorporate stress management and/or self-care for caregivers | - Relaxation exercises to be used by facilitators as-needed based on needs of caregivers the group |
| Caregivers | Improving physical space: Preference for a comfortable space that does not feel sterile or classroom-like, and making facial tissue available | - Moved group sessions to a more comfortable room - Placed facial tissue box on table |
| Caregivers & Youth | Improve explanations of content and organization of topics: Need to introduce content with clearer, more fulsome explanations, and in an order that enhances flow | - Reorganized content to ensure flow is logical based on feedback received - Increased clarity and thoroughness of explanations |
| Caregivers & Youth | Examples need improvement: Original examples were too simplistic and/or did not focus on issues that caregivers and youth perceived as relevant in the context of youth depression | - Reviewed all examples with youth advisors to enhance relevance (e.g., examples about keeping room clean changed to examples about not attending mental health appointments) |
| Caregivers & Youth | Need for flexibility and tailoring to needs of caregivers: Importance of understanding the challenges that caregivers in the group are supporting their child with, and using this information to inform content and examples | - Added more opportunities to solicit examples from caregivers for demonstration of strategies - Ensuring sufficient time for caregivers to raise issues or ask questions |
| Caregivers & Youth | Enhance psychoeducation about depression: Clearer and more thorough explanations of what depression looks like in young people, how it affects communication, and its causes and treatment | - Enhanced psychoeducation by incorporating content developed in our program’s integrated care pathway for youth depression (Courtney et al., 2021) - Revisited this information throughout later sections on communication and problem solving |
| Youth | Improving language: Clarifying language throughout; avoiding language of “positive” or “negative” emotions; referring to young people as “your child” instead of “teen” or “adolescent” | - Language modified throughout, as suggested |
| Youth | Informing child about group: Youth wanted to know their caregiver was participating in the program and would be trying new ways of communicating | - Encouraged caregivers in Session 1 to inform their youth that they are in the group, and followed up on this in Session 2 - Added notes to home practice suggesting caregivers inform youth that they would be trying a new way of communicating |
| Youth | Incorporate youth insights into communication suggestions: Importance of caregivers attending to youth cues about communication, offering support while allowing age-appropriate autonomy, providing validation, and responding appropriately to suicidal ideation | - Revised communication suggestions based on youth advisors’ input |

1. **Feedback That Did Not Result in Changes**

| Source | Feedback Theme | Rationale for Not Making Changes |
| --- | --- | --- |
| Caregivers | More of the program: More or longer sessions | Retained 8 session format to ensure feasibility of program based on clinical resources |
| Caregivers | Improve food: A light pizza snack was offered, and some caregivers did not enjoy it | Unable to change due to budget limitations |
| Caregivers | Scheduling challenges: Difficulty attending sessions in person due to work schedules etc. | Maintained 5pm start time due to staff availability; however, future versions of the program have been run via videoconference |
| Caregivers | Involving youth in sessions: We asked caregiver advisors if they would find it helpful to involve either their child or a youth advisor in the sessions (e.g., to practice skills) | Feedback from advisors was mixed so we did not pursue this further |
| Caregiver | Discussing medication: One caregiver requested that information on medication and managing its side effects be added to the program | Because we anticipate that the group will be facilitated by psychologists, social workers, or youth workers in the future, this would not be within their scope, so it was not included |
| Youth | Caregiver role in depression: Raising possibility of caregivers causing depression | This was not added because: 1) research evidence suggests the association between caregiver-youth relationship problems and youth depression is bidirectional (e.g., Hale et al., 2016); and 2) it would likely alienate caregivers, many of whom already feel guilty or to blame for their child’s depression (Stapley et al., 2016) |

Table S2

*Guidance for reporting intervention development studies in health research (GUIDED) Checklist* (Duncan et al., 2020)

| **Item description** | **Page in manuscript where item is located** |
| --- | --- |
| 1. Report the context for which the intervention was developed. | 6-7 |
| 2. Report the purpose of the intervention development process. | 6 |
| 3. Report the target population for the intervention development process. | 6-7 |
| 4. Report how any published intervention  development approach contributed to the development process | 7 |
| 5. Report how evidence from different sources informed the intervention development process. | 9-10 |
| 6. Report how/if published theory informed the intervention development process. | 8-9 |
| 7. Report any use of components from an existing intervention in the current intervention development process. | 7 & Table 1 |
| 8. Report any guiding principles, people or factors that were prioritised when making decisions during the intervention development process. | 7 |
| 9. Report how stakeholders contributed to the intervention development process. | 10-11 & Supplement pages 1-2 |
| 10. Report how the intervention changed in content and format from the start of the intervention development process. | Supplement pages 1-2 |
| 11. Report any changes to interventions required or likely to be required for subgroups. | 13 |
| 12. Report important uncertainties at the end of the intervention development process. | 13-14 |
| 13. Follow TIDieR guidance when describing the developed intervention. | Table S2 |
| 14. Report the  Intervention development process in an open access format. | Complete (no page location) |

Table S3

*Template for Intervention Description and Replication (TIDieR) Checklist* (Hoffmann et al., 2014)

| Item Number | Item | Page in Manuscript |
| --- | --- | --- |
| Brief Name | | |
| 1 | Provide the name or a phrase that describes the intervention | 1 |
| Why | | |
| 2 | Describe any rationale, theory, or goal of the elements essential to the intervention | Rationale: 4  Theory: 8-9 |
| What | | |
| 3 | Materials: Describe any physical or informational materials used in the intervention, including those provided to participants or used in intervention delivery or in training of intervention providers. Provide information on where the materials can be accessed (such as online appendix, URL) | 11-12 |
| 4 | Procedures: Describe each of the procedures, activities, and/or processes used in the intervention, including any enabling or support activities | 11-13 |
| Who provided | | |
| 5 | For each category of intervention provider (such as psychologist, nursing assistant), describe their expertise, background, and any specific training given | 12 |
| How | | |
| 6 | Describe the modes of delivery (such as face to face or by some other mechanism, such as internet or telephone) of the intervention and whether it was provided individually or in a group | 11 |
| Where | | |
| 7 | Describe the type(s) of location(s) where the intervention occurred, including any necessary infrastructure or relevant features | 7 |
| When and How Much | | |
| 8 | Describe the number of times the intervention was delivered and over what period of time including the number of sessions, their schedule, and their duration, intensity, or dose | 12 |
| Tailoring | | |
| 9 | If the intervention was planned to be personalised, titrated or adapted, then describe what, why, when, and how | N/A |
| Modifications | | |
| 10 | If the intervention was modified during the course of the study, describe the changes (what, why, when, and how) | Supplement pages 1-2 |
| How Well | | |
| 11 | Planned: If intervention adherence or fidelity was assessed, describe how and by whom, and if any strategies were used to maintain or improve fidelity, describe them | N/A |
| 12 | Actual: If intervention adherence or fidelity was assessed, describe the extent to which the intervention was delivered as planned | N/A |

Appendix A

Sample Caregiver Satisfaction Survey Items

**1. How would you rate the quality of the parent group?**

| *Excellent* |  | *Good* |  | *Fair* |  | *Poor* |
| --- | --- | --- | --- | --- | --- | --- |
| *4* |  | *3* |  | *2* |  | *1* |

**2. Overall, how satisfied are you with the parent group?**

| *Very*  *satisfied* |  | *Somewhat*  *satisfied* |  | *Somewhat dissatisfied* |  | *Very dissatisfied* |
| --- | --- | --- | --- | --- | --- | --- |
| *4* |  | *3* |  | *2* |  | *1* |

**3. Were the information and materials you received during the group sessions helpful?**

| *Yes, definitely* |  | *Yes, I think so* |  | *No, I don’t think so* |  | *No, definitely not* |
| --- | --- | --- | --- | --- | --- | --- |
| *4* |  | *3* |  | *2* |  | *1* |

**4. Have the skills you learned through the group helped you to deal more effectively with your child’s / youth’s mood difficulties?**

| *Yes, they helped a great deal* |  | *Yes, they helped somewhat* |  | *No, they really didn’t help* |  | *No, they seemed to make things worse* |
| --- | --- | --- | --- | --- | --- | --- |
| *4* |  | *3* |  | *2* |  | *1* |

**5. How satisfied are you with the way you have been treated by the group staff?**

| *Very*  *satisfied* |  | *Somewhat*  *satisfied* |  | *Somewhat dissatisfied* |  | *Very dissatisfied* |
| --- | --- | --- | --- | --- | --- | --- |
| *4* |  | *3* |  | *2* |  | *1* |
|  |  |  |  |  |  |  |

**6. How helpful did you find the following group sessions?** *Please circle* ***one*** *response for each of the following topics….*

| **Topic** | *Very*  *helpful* | *Somewhat helpful* | *Not very helpful* | *Not at all helpful* |
| --- | --- | --- | --- | --- |
| **About Depression** | *4* | *3* | *2* | *1* |
| **Cognitive Behavioural Model** | *4* | *3* | *2* | *1* |
| **Active Listening** | *4* | *3* | *2* | *1* |
| **Stating Positive & Negative Feelings** | *4* | *3* | *2* | *1* |
| **Problem Solving Part 1** | *4* | *3* | *2* | *1* |
| **Problem Solving Part 2** | *4* | *3* | *2* | *1* |
| **Practice & Implementation** | *4* | *3* | *2* | *1* |
| **Review and Conclusions** | *4* | *3* | *2* | *1* |

**7. Please tell us what you liked the MOST about the parent/caregiver group?**

**8. Please tell us what you liked the LEAST about the parent/caregiver group?**

**9. Please share your suggestions for improving our parent/caregiver group:**
